# Ethnographic study of the use of interventions during the second stage of labor in Jeddah, Saudi Arabia

**DOI:** 10.1111/birt.12395

**Published:** 2018-09-09

**Authors:** Roa Altaweli, Christine McCourt, Mandie Scamell, Katherine Curtis Tyler

**Affiliations:** ^1^ East Jeddah Hospital Jeddah Saudi Arabia; ^2^ Department of Maternal and Child Health, School of Health Sciences City, University of London London UK; ^3^ School of Health Sciences City, University of London London UK

## Abstract

**Background:**

Routine use of medical interventions during labor has been identified as a clinical area for concern, since such routinized practice is not consistent with an evidence‐based approach to care and continues to increase despite efforts to encourage normal childbirth. Therefore, the aim of our study was to explore maternity health professionals’ use of interventions during the second stage of labor in two hospitals in Jeddah, Saudi Arabia, to understand what influences their decision‐making and practices.

**Methods:**

This was an exploratory study using an ethnographic approach. Data collection methods included participant observations of 19 labors and births (n = 8 at City Hospital and n = 11 at King's Hospital) and semi‐structured interviews with 29 health care professionals. In addition, the hospital labor and delivery ward policies and guidelines from those hospitals were collected. Data were analyzed thematically.

**Results:**

Medical interventions were used during the second stage of labor routinely, regardless of clinical indication. Three core influences that shaped the clinical decision‐making were identified as follows: (a) organizational culture, (b) a medical concept of birth, and (c) a hierarchical system of control. We suggest that the clinical decision‐making and routine practice in this setting arises out of the interface between these three core influences whereby hierarchical control and clinicians’ exercise of power and feelings of powerlessness are fundamental drivers for an organizational culture of medicalized childbirth, despite the differing models of childbirth which professionals described.

**Conclusions:**

Clinical decisions relating to the use of interventions during childbirth are both complex and socially negotiated. The findings reflect the complexity of the use of interventions during the second stage of labor and the multiple influences on professionals’ practices. We have shown how three key influences interact to shape clinical decision‐making during the second stage of labor in this cultural setting and how the use of medical interventions can be analyzed as an illustration of the power dynamic in the maternity health care system. We suggest that written policies are insufficient to bring about evidence‐based practice and approaches to change need to take into account these different levels of influence.

## 1 INTRODUCTION

Routine use of medical intervention during labor has been identified as a clinical area for concern, since such routinized practice is not consistent with an evidence‐based approach to care.^1^ The adverse consequences of many interventions when used routinely are well documented and guidelines such as the United Kingdom NICE guidelines on intrapartum care,^2^ based on systematic evidence reviews, recommend that if labor is progressing normally and both mother and baby are well, clinical interventions should not be offered or advised. However, studies have shown that many women with straightforward pregnancies are subject to a range of medical interventions during labor and birth in a range of countries, including Saudi Arabia.^3^, ^4^, ^5^, ^6^, ^7^, ^8^, ^9^, ^10^, ^11^ Practices include the unnecessary use of continuous electronic fetal monitoring (EFM), lithotomy positioning, episiotomy, directed pushing, instrumental deliveries, and artificial rupture of the membranes.^3^, ^4^, ^5^, ^6^, ^7^, ^8^, ^9^ Furthermore, research in Saudi Arabia has revealed that such interventions in childbirth are on the rise.^12^

In *The Lancet* Maternal Health Series, Miller et al^11^ examined two extremes in global maternal health care: too little, too late and too much, too soon. Too much, too soon describes the routine overmedicalization of normal pregnancy and birth resulting in unnecessary interventions. As hospital births increase, so does the recognition that too much, too soon causes harm and increases health costs, and often concentrates disrespect and abuse. Health professionals and health systems need to ensure that all women receive high‐quality, evidence‐based, equitable, and respectful care. In Saudi Arabia, over 90% of births take place in hospitals, and there are few other birthing options.^13^

This article describes the findings of an ethnographic study examining the use of routine interventions during the second stage of labor in Jeddah, Saudi Arabia. Concern about the iatrogenic risks associated with the increasing routine medicalization of childbirth in Saudi Arabia, and the urgency to understand the influences that drive this increase, underpinned the rationale for the study. In addition, the authors chose to focus on the second stage of labor, as the routine use of interventions at this stage is relatively understudied, as compared with first stage interventions.

The purpose of this study was to investigate the perceptions, attitudes, and practices of obstetricians, midwives, and nurses caring for laboring women in two different maternity care units in order to understand what influences their decisions to use interventions during labor. In this article, we acknowledge the physiological potential for childbirth to be a spontaneous biological event, and that this event is culturally informed.^14^ That is to say, the study rests upon an understanding that birth practices are context bound and are constituted through both social and biological influences.^15^

## 2 METHODS

Using an ethnographic approach, this study focussed upon two large public hospitals providing maternity care in Jeddah, Saudi Arabia. Ethnographic fieldwork was carried out by the first author (RA) between October 2011 and September 2012. One of the hospitals included is regulated by the Ministry of Health (City Hospital—pseudonym) and the other is regulated by a military government body (King’s Hospital—pseudonym). The choice of research sites reflects the diversity of public maternity care provided in Jeddah.

Data collection methods included participant observations of 19 labors and births—8 at City Hospital and 11 at King's Hospital—and semi‐structured interviews with 29 health care professionals from the two hospitals in Jeddah. In addition, the hospitals’ labor and delivery ward policies and guidelines were collected and analyzed and a field diary was maintained. This use of multiple sources for data collection methods strengthened the study design and enabled a more comprehensive account of the context and use of interventions during the second stage of labor. The first author role as a researcher was that of a “participant observer.” She did not have any clinical duty and was not involved in clinical decision‐making, but she offered help with practicalities if required (translations between professionals and women, and getting teas and hot milk to women after birth) in order to “fit” into the scene without disturbing it.^16^

All interviews were tape‐recorded, with the permission of participants, using an Olympus WS 650S DNS digital voice recorder machine. The interviews were conducted using a semi‐structured interview topic guide (available on request). The interview focus progressed from description of usual practices through to professionals’ perspectives and opinions on the reasons for use and then their feelings about these practices.

Purposive sampling was used to recruit professional participants working on the labor ward directly with women during labor and birth. The sampling technique ensured access to interviewees able to provide in‐depth and appropriate data based on their experience with the key concept being explored.^17^ This sampling included obstetricians (n = 10), nurses (n = 6), midwives (n = 12), and nurse‐midwife (n = 1). The approach was supplemented with convenience sampling to enable observation of care in the maternity care units. It was clear that the labor ward is always busy, so interviews were conducted with the consenting professionals at a time when they had no woman in labor to care for or they were otherwise free to be interviewed.

Inclusion for women participants involved in the observation was restricted to low‐risk women defined as singleton, term pregnancy with vertex presentation. Women who became high‐risk during the observation, which commenced in active first stage labor, were subsequently excluded.

All available hospital policies and guidelines on second stage practices at King's and City Hospitals were collected for analysis. These were triangulated with findings from the interview and observation data, prompting further lines of inquiry that helped identify contradictions between written policies and observed practices or staff descriptions of policies that would not otherwise have been apparent.

Observation records, interview verbatim transcripts, field diary, and hospital documents were transferred into qualitative data analysis software (Atlas.ti 7) which was used for organizing and coding the data. The six phases of thematic analysis proposed by Braun and Clarke^18^ were used for the data analysis but based on a framework proposed by DeVries et al^19^ that distinguishes the macro‐, meso‐, and microlevels of analysis to take account of how maternity care is designed and shaped at different levels of society. Micro‐ and mesolevel analyses are relatively descriptive in nature. The macrolevel analysis illuminates the core themes that emerged during data analysis and provides a more explanatory focus. This approach provided a new way of conceptualizing the culture of maternity wards where interventions are used routinely during the second stage of labor.

### 2.1 Ethical considerations

Ethical approval from both City University's Research Ethics Committee on the 29.06.2011, Ref: PhD/10‐11/07 and from each of the hospitals involved in the study was granted prior to data collection.

All professionals working in labor and delivery ward in both hospitals were informed about the study and invited to participate. Information sheets and consent forms were placed in the staff pigeonholes on the ward. In addition, they were distributed personally by the researcher (RA) and by the nurse managers at each of the sites. Written consent was obtained either at the start of each shift, following the handover, or immediately before the interview or observation. Consent was reconfirmed verbally at the start of each observation or interview.

Women's consent for the observations was sought at diagnosis of early active labor (with 3‐6 cm cervical dilatation). The purpose of the study and the focus on the professional practices were explained, and the women were asked to sign a consent form if they were willing for their labor and birth to be observed. Interviews with women were not planned for this study, since the primary focus was on observing routine professional practices and professionals’ understandings of the reasons for these.

During the observations, an unobtrusive approach was taken. The researcher, a registered midwife, wore scrub of a different color to distinguish her researcher position; staff tended to view her presence in the room as similar to that of a typical midwifery student, although she did not engage in any clinical care.

Anonymity of the hospitals where recruitment took place was achieved by not reporting hospitals’ or participants’ real names in this study; only codes or pseudonyms were used. The research Id was designed to help categorizing the participants and providing quick information about them and to distinguish between quotations from different types of participants; the following codes were used: observation field note (O), obstetrician (OB), and midwife (MW). No identifying person data were included in data transcripts.

## 3 RESULTS

**Figure 1:**
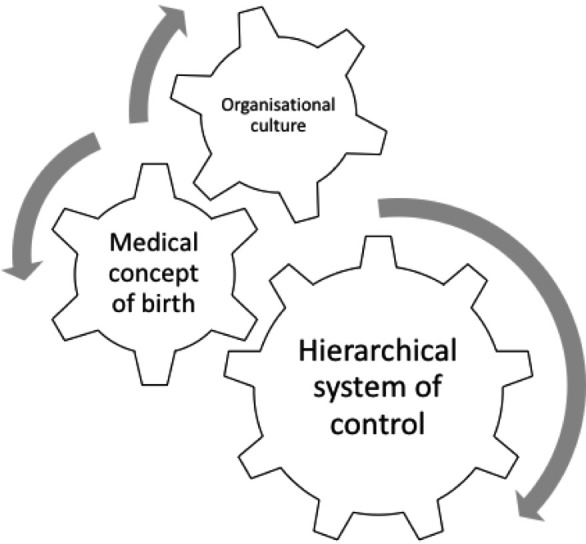
Core themes

Medical interventions were used during the second stage of labor routinely, regardless of clinical indication. Three core influences that shaped the clinical decision‐making were identified as follows: (a) organizational culture, (b) a medical concept of birth, and (c) a hierarchical system of control. We suggest that the clinical decision‐making and routine practice in this setting arises out of the interface between these three core influences. Figure 1 shows how hierarchical control and clinicians’ exercise of power and feelings of powerlessness is a fundamental driver for an organizational culture of medicalized childbirth, despite the differing models of childbirth which professionals described.

We set out these key findings below in three stages: First, a descriptive analysis drawing particularly on observation provides direct evidence of how organizational culture and concepts of birth are materialized in the labor environment—that is, how the design of the environment and ways of managing birth materially shape everyday practices; second, analysis of the dominant and muted concepts of birth held by professionals; and third, analysis of a how a hierarchical system of control influences practices.

### 3.1 Setting the scene—the birthing environment and observed practice

Observational data revealed that birth took place within a clinical environment/setting that was sterile, possibly uncomfortable for laboring women, bright, noisy, crowded, and cold during labor and birth. The hospital delivery beds in both hospitals were centrally placed in the labor rooms and were generally of obstetric design aimed to assist different interventions and procedures during labor and birth, and use of the lithotomy position. The labor and delivery rooms were clinical and mechanical in appearance with minimal furniture, metal surfaces that are easy to clean, a baby resuscitation bed, a cardiotocography (CTG) machine, theater lights, and stainless steel trolleys with a delivery pack on them. The available space left in the room was confined, providing women with little opportunity to move around or to feel comfortable. The birthing environment of both hospitals was crowded with not only machines but staff coming in and out, and the soundscape was dominated by the thumping of the EFM machines and continuous staff discussions.

The observations in both hospitals showed that during the active first stage until the end of the third stage of labor, women were not allowed to walk around. Routine use of continuous EFM meant that the women were confined to bed once they were diagnosed as being in active labor:

Sarah (a pseudonym) was lying on the bed on her left side, covered by a sheet. Since admission to the labour room, she had been attached to a continuous CTG machine by two wires, one to record the fetal heart rate and one to record the contractions. King’s Hospital‐O‐02

This confinement was exacerbated by the limitation of space which prevented women from mobilizing freely. Some women were allowed to walk around temporarily to go to the toilet, but in many cases, bladder catheters and intravenous fluids were attached meaning that their movements were even more restricted. The majority of these procedures were performed without seeking the women's consent.

The labor room in this study is designed to facilitate the use of interventions during labor. It is replete with technology, for example, CTG machines, intravenous units, automatic beds, and baby resuscitators. This clinical setting makes the woman a “guest in the house of medicine.”^20^ All the applications, uniforms, and medical explanations make it clear that science, medicine, and doctors dominate this setting.

Professionals interviewed in both hospitals stated that routine continuous use of CTG for EFM is hospital policy:

Because it is hospital policy, once someone is in active labour we need to put her on the CTG and monitor the foetal heart rate every 15 minutes. King’s Hospital‐SN1‐10

I think this is the protocol which is followed here in the hospital, especially if the patient is in active labour. Then we have to do continuous foetal monitoring. City Hospital‐OB‐01

However, King's Hospital's written policy recommends using intermittent auscultation with a Pinard Stethoscope or Sonicaid for low‐risk patients:

Foetal monitoring of low‐risk patients may be by auscultation with a Pinnard Stethoscope or Sonicaid for one minute…The CTG trace may be intermittent in low‐risk pregnancies, to allow for review of the foetal heart trace combined with more mobility for the patient in labour…Studies show that intermittent auscultation for low‐risk patients has no detrimental effect, as the outcome was the same as it would have been if they had been monitored continuously by CTG monitoring… King’s Hospital fetal monitoring policy

Despite the hospital policies and guidelines, intermittent auscultation was not an option in the hospitals involved in the study and we did not observe any birth where professionals used the CTG machine intermittently.

### 3.2 Concepts of birth: dominant and muted understandings

This section builds on the birthing space description above by providing insight into the justifications and explanations professionals gave for using interventions during labor and birth and what this revealed about their concepts of birth. A medicalized model of childbirth was observed to guide practice in both hospitals and in the interviews, while interviews illuminated that within this highly medicalized environment, many professionals appeared to devalue their own skills and knowledge about healthy birthing and defer to the dominant culture of medicalized birth.

Professionals’ views of birth in this study are complex and mixed. While some of the participants openly expressed positive views and attitudes toward the use of interventions during their interviews, others had more critical views or no opinion. In this context, the routine application of medical surveillance and intervention in low‐risk birth could be interpreted by interviewees as noninvasive and an indication of excellent practice, as one obstetrician explained:

… In my experience at King's Hospital, all our interventions are in the second stage … I'm happy with these interventions because they are successful. Occasionally there are complications … During management of the second stage, we are over‐caring towards the patient. In other hospitals, although they manage them there is not continuous follow‐up or close observation as there is here. King’s Hospital‐OB‐15

However, some of the professionals interviewed also argued that birth should be viewed as a natural healthy event. For example, one obstetrician expressed the belief that women should progress naturally and argued that professionals intervene too much:

Sometimes I feel that we intervene too much given that childbirth is a natural process and that maybe if we just let the patient be and let her progress on her own without too much intervention, it would be better than all the things we are doing. For example, we do not have to encourage the patient to push as soon as she is fully dilated. We should just let the patient progress on her own. The other thing is, for example, stretching of the perineum and massaging it. Again, I would rather the patient did it on her own. So, I feel that we're intervening too much and at the end of the day, it's just a natural process. King’s Hospital‐OB‐13

A few midwives and obstetricians, in their interviews, acknowledged the psychological and social aspects of giving birth, including building trust between the midwife and the woman and providing reassurance, as this quotation shows:

Of course she [the woman] is the main actor in this movie … because you see…. the woman will give you good results in the end… I believe the midwife should be very close to her patient. I mean the patient should feel protected and safe with the nurse, so everything proceeds smoothly, with no complications. King’s Hospital‐MW‐04

In addition, an obstetrician stated that they should decrease intervention to make everyone relaxed:

Especially with low‐risk patients, if we decreased our intervention it would be relaxing for me, for the doctor, for the staff, the nurse, and even for the patient. King’s Hospital‐OB‐15

However, this model of birth did not seem to influence their observed clinical decision‐making. There was no evidence from the observation of the coexistence of different ways of knowing about childbirth in practice.

### 3.3 Hierarchical control: clinicians’ exercise of power and feelings of powerlessness

In both hospitals, during the course of the observations and interviews, it appeared that professionals hold limited power or perceive their power as limited in the face of “hospital protocols and guidelines.”

A sense of powerlessness appeared to pervade professionals’ clinical decision‐making and their accounts of this. Following what they perceived to be hospital rules reduced clinical autonomy for all groups of professionals involved in this study, in addition to constraining the autonomy of women in labor. Obstetricians, midwives, and nurses alike, all described having their clinical discretion impeded by “rules.” As one obstetrician described:

The department decides on a certain pathway to manage the second stage of labour. This is then something we all have to follow. King’s Hospital‐OB‐13

However, as described in the previous section, analysis of the written clinical guidelines identified that many practices that professionals considered to be protocol‐based were not in accord with the written guidelines. This suggests that “the department” is understood as having independent autonomy to exercise control, which appears to exist over and above the individual members of that department. The auspices of this apparently free‐floating “medical control” meant that laboring women could not be left to birth spontaneously without medical surveillance and intervention:

What is the benefit of becoming a doctor or obstetrician and then leaving the patient to deliver on her own? … So we don't — we are not allowed to leave the patient to deliver naturally without anything. And that's why obstetricians work in the delivery room. King’s Hospital‐OB‐15

Observational data from the study revealed that the obstetricians act as gatekeepers, being informed, giving orders, and permitting midwives, at times (such as when busy), to lead care. This does not mean that doctors consistently pursue the medicalization of birth and midwives resist it. For example, one midwife stated with resignation:

It’s all by doctor's order, I cannot do any intervention without the doctor’s order. Just a normal delivery. Even a midwife case, even a nurse she cannot administer any medication without a doctor’s order. City Hospital‐MW‐03

Midwives expressed feeling powerless to manage care on their own professional judgment and being controlled by the doctors’ preferences, but they were also observed to draw heavily on the medical model of birth to ensure that they maintained power over the women in their care.

In some cases, midwives attempted to exert power over both women and doctors, but they could also feel sandwiched between them:

First of all, sometimes we deal with an uncooperative patient and secondly, some doctors give us a lot of orders while we are very busy with a patient. They give orders, and you know how it is when there are 3 or 4 doctors in the room, and that affects the delivering midwife, in contrast to when you are delivering alone in the room and you know exactly what are you doing. City Hospital‐MW‐07

It was not surprising therefore that some midwives and nurses described feeling powerless during labor and birth. As this midwife explained, this sense of powerlessness can build up resentment between professional groups:

We need permission from the doctor. This unit is sort of medically managed because if you are a midwife, whatever you do, you are still under the doctor's control … You find that junior doctors are supervising each other in medical cases and also the medical staff. It's as if they do not consider you as an experienced person when you are there. Even if I explain something, they listen to their own superior and not to the midwife, and then something goes wrong. King’s Hospital‐MW‐01

Pain, an inevitable part of childbirth, was described as unacceptable. Medical intervention within this context was perceived as protecting women from pain:

The second thing for me, after a good outcome and the well being of the mum and baby, is the pain. No one likes to see someone in pain, so we have to interfere, especially during the second stage. King’s Hospital‐OB‐15

However, when interventions introduced by professionals themselves led to increased pain for the woman, this was usually ignored. For example, during the observation of birth 4 at King's Hospital:

The midwife changed the woman from the right lateral to left lateral position. The woman said “I don't feel comfortable like this”. The midwife ignored her and kept her in that position. King’s Hospital‐O‐04

Such failure of professionals to take the women's feelings or expressed wishes into consideration during the management of the second stage was common. During one of the observations, for example, a midwife inserted a urinary catheter against the woman's wish, causing additional pain and without her consent:

The midwife was preparing a sterile (in and out) urinary catheter to empty the bladder … She told the woman:

“I will remove the urine now”.

The woman replied, “I want to go to the toilet”.

The midwife said: “You will push your baby in the toilet”.

The woman said: “I promise you that I will not push the baby in the toilet”.

The midwife ignored her and continued to insert the catheter and asked the woman to take a breath.

The woman said: “I want to go to the toilet please! I don't want the tube. I want to go to the toilet”. The urinary catheter was inserted. King’s Hospital‐O‐04

Whereas professionals reported their own felt lack of power and authority in the system, the observations highlighted that women were below professionals in this hierarchy, with little attempt given to inform the women about care practices or to seek their consent for them. Within this hierarchy, women are expected not to interfere with medical practice and decision‐making. Instead, they were expected to comply and co‐operate. As one of the obstetricians stated:

Medically, she's not allowed to interfere with our medical decisions at that point. I don't mean she's not allowed, but we need to explain all the complications to her and we need her to agree with what we are doing, and if it is not agreed and the problem happens, she will not stop blaming herself. So, it's better to explain that thoroughly to her and then finally we follow our decision rather than hers. King’s Hospital‐OB‐09

This kind of paternalistic approach to care, where women were relieved of the responsibility of autonomy, rendered them passive in their own birth.

Although most of the professionals understood the consequences of the natural and medical models of childbirth, their thinking, their accounts, and our observations suggest that they dismissed the natural model to follow what they perceived to be hospital policy, including to deal with their fears of medicolegal problems or undefined sanctions. For example,

In normal deliveries there shouldn’t be interventions, or at least they should be kept to a minimum, but medico‐legal problems make us interfere sometimes when we shouldn’t do so. I feel the less the interventions the better the outcome. City Hospital‐OB‐12

They felt themselves limited by their position in the hospital hierarchy, and their feelings of impotence to challenge institutional power. A professional hierarchy was clearly present, with doctors at the top of the hierarchy, followed by midwives and then by nurses, and finally the women. Yet even those at the top of the medical hierarchy such as senior obstetricians described being constrained in this way. For example, one senior obstetrician stated:

In our centre we don't allow the second stage to go on longer than two hours. It's difficult for me to break this rule. I know I could; it's doable, but it's not in the books. It's done sporadically, it's not witnessed or protected by any of the literature, so I don't do it. King’s Hospital‐OB‐12

## 4 DISCUSSION

This study forms part of a global discussion on labor ward culture. Ethnographic research and use of observations is an increasingly popular approach in midwifery research in the labor room. Several authors have used ethnographic research to investigate the culture within labor rooms.^21^, ^22^, ^23^, ^24^, ^25^, ^26^, ^27^, ^28^, ^29^, ^30^, ^31^, ^32^ Other studies have used observation in the labor room without an explicit ethnographic approach.^33^, ^34^, ^35^ The study findings have demonstrated that although professional views are complex, a medical model of birth prevails, where interventions in the second stage of labor are practiced routinely without consideration of clinical indication or the needs and wishes of birthing women. Medical models of childbirth have become dominant in most countries, as evidenced by the increase in cesarean delivery rates worldwide, a factor that is transforming the nature of childbirth.^36^ This is in line with a worldwide shift from home births to hospital birth, and more women giving birth within a hospital setting.^37^ This research demonstrates that when practiced in this way, the interventionist approach to care becomes the norm and professionals may fail to perceive it as either invasive or as having iatrogenic risks, or even if they do perceive this, their perceptions may become muted and subordinate to a normative medical model.^38^

Although some of the health care professionals involved in this study did talk about the importance of the psychological and social aspects of giving birth, including providing reassurance and building trust between the midwife and the woman (often derived from practice experiences in other national settings), they continued to follow the medical model and the social/midwifery model was mainly absent in their practice. All professionals observed during this study overwhelmingly *treated* birth as a medical problem rather than a biopsychosocial event and transition. The organizational culture shaped the birthing environment and routine practices in a hierarchical context that profoundly influenced clinical decision‐making during the second stage of labor.

There is no doubt that medical interventions in the second stage of labor can be life‐saving when clinically indicated. When used routinely, however, clinical judgment becomes irrelevant, meaning that adverse consequences such as distress, pain, or morbidity to mothers and babies^39^ are overlooked and interventions may become a source of harm rather than benefit. For an intervention to be “necessary,” they must be based on evidence, do more good than harm, and should not be used routinely.^39^, ^40^ In an effort to address this issue, the World Health Organization has produced new recommendations on intrapartum care that promote more women‐centered care and avoidance of routine interventions.^41^

The distinction between an interference in what can be understood as an otherwise spontaneous, physiological event and what is a necessary intervention to prevent morbidity and mortality in the second stage of labor, rests upon professional discretion underpinned by robust research, practice experience, and informed choice and consent of the patient, a combination which is otherwise known as evidence‐based practice.^42^, ^43^, ^44^ The evidence on the use of medical interventions during the second stage of labor is conclusive in demonstrating that selective use is optimal.^45^ The findings from this study, however, indicate that even when hospitals update their clinical guidelines to discourage the routine medicalization of birth, a series of “soft” rules may operate that are so deeply embedded and embodied in the everyday clinical decision‐making during the management of labor that the opportunity for professional discretion is rarely articulated. By “soft rules” we mean that these rules are rather abstract, not based on guidelines that are written down, which in this setting were more evidence‐based.

Professionals trained in a hierarchical and medicalized health system may fear that they cannot work outside the hospital's soft rules and argue that hospital policymakers have the ultimate power. Following such rules, moreover, reduces power across professional boundaries, rather than only affecting midwives or nurses. It suggests that power operates over and above any of the specific social groups involved but also involves denial or lack of acknowledgment of the powers that professionals do exercise. Institutional anxiety is prevalent, limiting autonomy among professionals because of an unwillingness not to adhere to the perceived rules. Technological advances in maternity care, when used selectively, can improve outcomes for both the mother and the neonate and enhance professional satisfaction with their work. When used indiscriminately, however, they can become a technique for oppression for health professionals and women alike.

### 4.1 Strengths and limitations

To our knowledge, this is the first ethnographic research conducted in the labor room in Saudi Arabia. It is also the first to document the interventions used during the second stage of labor and the reasons for their use in this context. This study has some limitations. This research did not explore the perceptions of women during childbirth but instead explored professionals’ actions and opinions, focusing on their perceptions, and observing their practices to uncover aspects of obstetric and midwifery culture in labor and delivery units. However, a strength was that observation was used to document practices and indeed included women's responses to these, enabling professional assumptions about women's preferences to be challenged.

### 4.2 Conclusions

This ethnographic study provided an opportunity to explore the use of interventions during the second stage of labor among professionals in Jeddah, Saudi Arabia. The findings reflect the complexity of the use of interventions during the second stage of labor and the multiple influences on professionals’ practices. Clinical decision‐making relating to the use of interventions during childbirth is both complex and socially situated and negotiated. This ethnographic study found that written policies were insufficient to bring about evidence‐based practice. We have shown how three key influences—the organizational culture, the prevalence of the medical model, and a rigid power hierarchy—interact to shape clinical decision‐making during the second stage of labor in this cultural setting and how the use of medical interventions can be analyzed as an illustration of the power dynamic in the maternity health care system.
